# Supplementary material for: Long-Acting FGF21 Inhibits Retinal Vascular Leakage in In Vivo and In Vitro Models
Source: Int J Mol Sci. 2020 Feb 11;21(4):1188. doi: 10.3390/ijms21041188 (PMC7072824; doi:10.3390/ijms21041188)
Supplement: Supplementary file 1 [file ijms-21-01188-s001.pdf]

Supplemental figure 1. The mRNA level of crucial receptors of FGF21

Level of mRNA expression of *FGFR1*(A), *FGFR2* (B), *FGFR3* (C), *FGFR4* (D), and *β-KLOTHO* (E) in human (h) VEGF-treated primary human retinal microvascular endothelial cells (HRMECs).

Supplemental figure 2. FGF21 preserves *CLDN-1* mRNA expression in HRMEC

qRT-PCR analysis of effect of FGF21 on *CLDN1* (A), *CLDN5* (B), *VE-CADHERIN* (C), and *OCLN* (D) in the HRMECs. Samples were collected 6 hours after hVEGF induction. n.s., not significant. Fold change was calculated (n = 6, * $p < 0.05$).

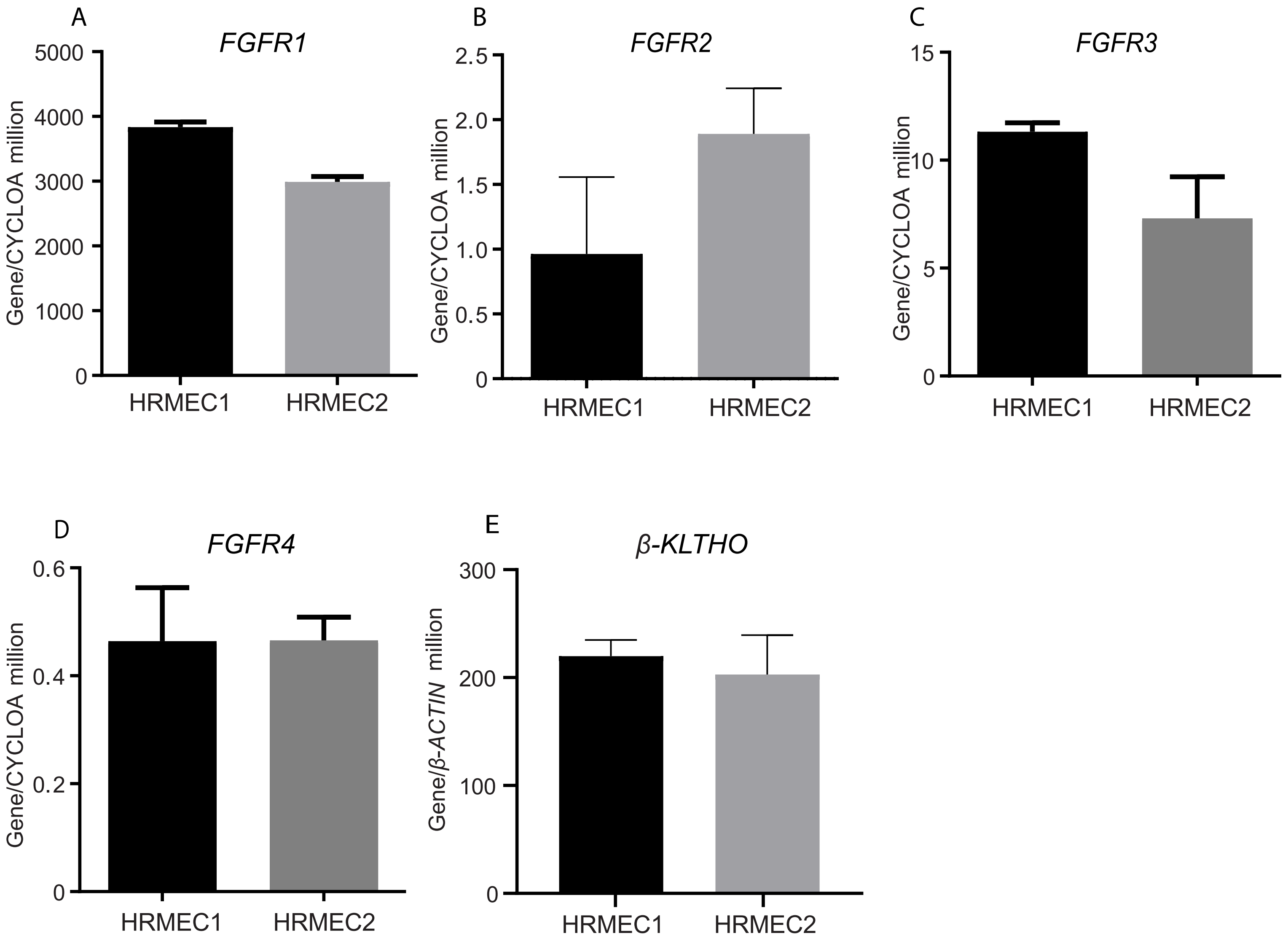

Tomita et al. Supplemental Figure 1

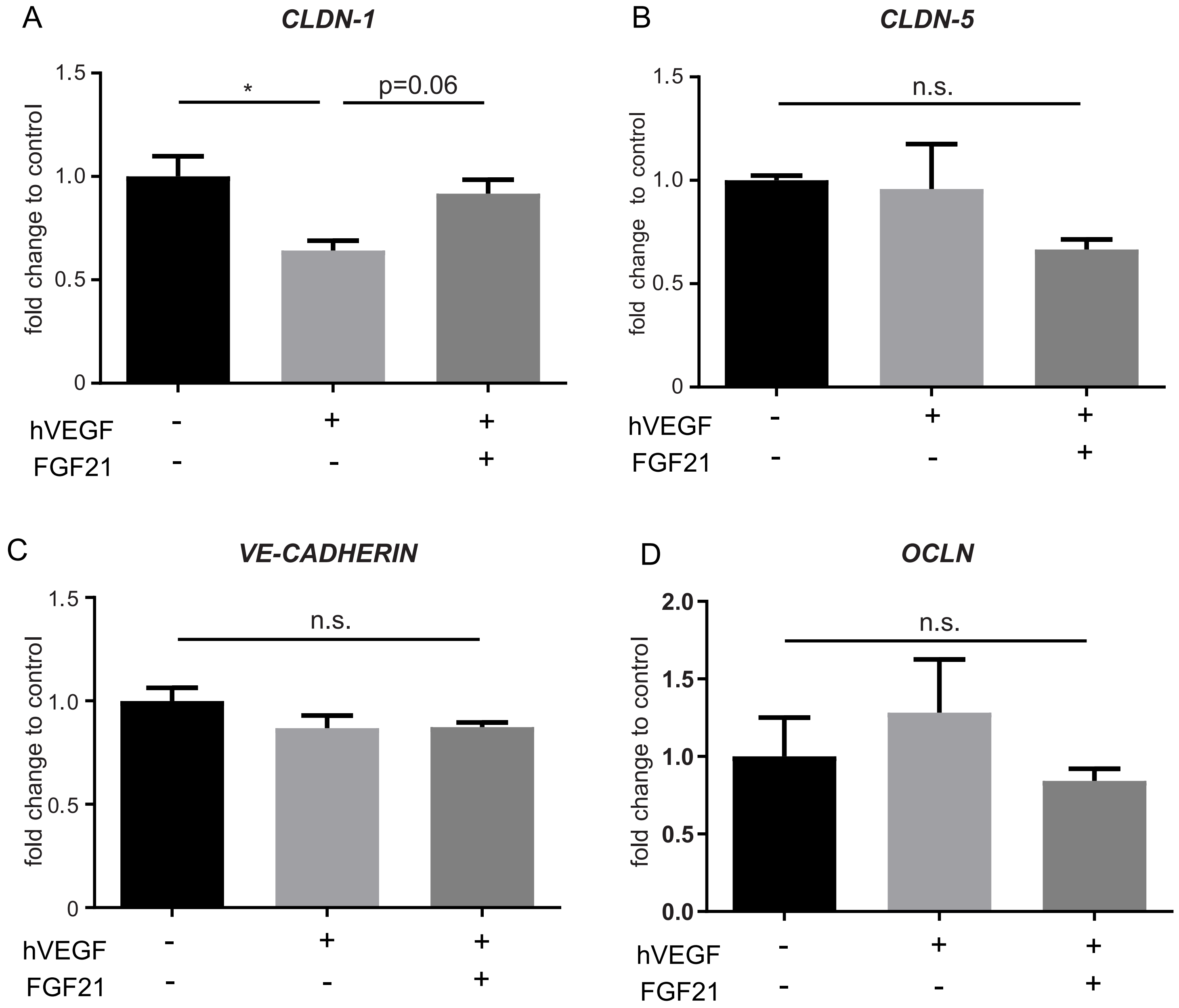

Tomita et al. Supplemental Figure 2

Supplemental table 1.

Primers list for RT-PCR

Gene	Primers
18S-F	5’-ACGGAAGGGCACCACCAGGA -3’
18S-R	5’- CACCACCACCCACGGAATCG-3’
CLDN-1-F	5’- GCATGAAGTGTATGAAGTGCTTGGA-3’
CLDN -1-R	5’- CGATTCTATTGCCATACCATGCTG-3’
CLDN -5-F	5’- CTCTGCTGGTTCGCCAACA-3’
CLDN -5-R	5’- CCCAGCTCGTACTTCTGTGACA-3’
VE-CADHERIN-F	5’- TTGGAACCAGATGCACATTGAT-3’
VE-CADHERIN-R	5’- TCTTGCGACTCACGCTTGAC -3’
OCLN-F	5’- ACAAGCGGTTTTATCCAGAGTC-3’
OCLN-R	5’- GTCATCCACAGGCGAAGTTAAT-3’
FGFR1-F	5’- CCCGTAGCTCCATATTGGACA-3’
FGFR1-R	5’- TTTGCCATTTTTCAACCAGCG-3’
FGFR2-F	5’- AGCACCATACTGGACCAACAC-3’
FGFR2-R	5’- GGCAGCGAAACTTGACAGTG-3’
FGFR3-F	5’- CCCAAATGGGAGCTGTCTCG-3’
FGFR3-R	5’- CCCGGTCCTTGTCAATGCC-3’
FGFR4-F	5’- CCATAGGGACCCCTCGAATAG-3’
FGFR4-R	5’- CAGCGGAACTTGACGGTGT-3’

β-KLOTHO-F	5’- TTCTGGGGTATTGGGACTGGA-3’
β-KLOTHO-R	5’- CCATTCGTGCTGCTGACATTTT-3’
CYCLOPHILIN-A-F	5’-CCCACCGTGTTCTTCGACATT-3’
CYCLOPHILIN-A-R	5’-GGACCCGTATGCTTTAGGATGA-3’
β-ACTIN-F	5’- AGAGCTACGAGCTGCCTGAC-3’
β-ACTIN-R	5’- AGCACTGTGTTGGCGTACAG-3’
